# Potential anti-osteoporotic effects of herbal extracts on osteoclasts, osteoblasts and chondrocytes *in vitro*

**DOI:** 10.1186/1472-6882-14-29

**Published:** 2014-01-17

**Authors:** Yoshiki Mukudai, Seiji Kondo, Tomoyuki Koyama, Chunnan Li, Seika Banka, Akiko Kogure, Kazunaga Yazawa, Satoru Shintani

**Affiliations:** 1 Department of Oral and Maxillofacial Surgery, School of Dentistry, Showa University, 2-1-1 Kitasenzoku, Ota-ku, Tokyo 145-8515, Japan; 2 Laboratory of Nutraceuticals and Functional Foods Science, Graduate School of Marine Science and Technology, Tokyo University of Marine Science and Technology, 4-5-7 Konan, Shinagawa-ku, Tokyo 108-8477, Japan; 3 Medical College, Jinggangshan University, Ji’an, Jiangxi Province 343009, P.R. China

**Keywords:** Herbal extract, Osteoporosis, Osteoclast, Osteoblast, Shondrocyte, Traditional Chinese medicine

## Abstract

**Background:**

Osteoporosis (OP) is one of the most serious diseases in the modern world, and OP patients frequently suffer from fragility fractures in the hip, spine and wrist, resulting in a limited quality of life. Although bisphosphonates (BPs) are the most effective class of anti-bone-resorptive drugs currently available and the most commonly prescribed for the clinical treatment of OP, they are known to cause serious side effects such as bisphosphonate-related osteonecrosis of the jaw. Novel therapeutic materials that can replace the use of BPs have therefore been developed.

**Methods:**

We commenced an institutional collaborative project in which candidates of herbal extracts were selected from more than 400 bioactive herbal products for their potential therapeutic effects not only in OP, but also in oral and skeletal diseases. In the present study, we report on 3 Chinese medical herbal extracts from the root barks of *Melia azedarach*, *Corydalis turtschaninovii*, and *Cynanchum atratum*.

**Results:**

All of these extracts inhibited osteoclast proliferation and induced apoptosis by up-regulation of caspase activity and increase of mitochondrial pro-apoptotic proteins expression. Furthermore, the extracts enhanced differentiation, but did not affect proliferation of both osteoblasts and chondrocytes. The osteo-inducible effect was also observed in cultured primary bone marrow cells.

**Conclusions:**

Although these extracts have been utilized in traditional Chinese medicine for hundreds of years, there are no reports to our knowledge, on their therapeutic effects in OP. In this study, we elucidate the potency of these herbal extracts as novel candidates for OP therapy.

## Background

Osteoporosis (OP) is one of the most serious diseases in the modern world, and OP patients frequently suffer from fragility fractures in the hip, spine and wrist, resulting in a limited quality of life. OP is often observed in elderly people, particularly postmenopausal women, and is caused by an imbalance between bone formation and bone resorption (reviewed in ref. [1-3]). Bisphosphonates (BPs) are the most effective class of anti-resorptive drugs currently available and are frequently used in the clinical treatment of OP, owing to the great advantages. Despite showing clinically beneficial effects in the treatment of OP, serious side effects of BPs have been reported, including bisphosphonate-related osteonecrosis of the jaw (BRONJ) [4]. Medical practitioners and basic researchers have therefore focused on the use of novel therapeutic materials such as anabolic and hormone-like agents that can replace and/or reduce the use of BPs.

In recent decades, Chinese medical herbal extracts have been extensively investigated for their effects on proliferation and differentiation of osteoclasts (OCs) and osteoblasts (OBs) *in vitro*, and/or therapeutic potency in OP *in vivo* (*e.g., Cistanche salsa*[5], *Anoectochilus formosanus*[6], *Acanthopanax senticosus*[7], *Herba Epimedii*[8] and *Curcuma longa*[9-12]). In order to identify more candidates of herbal extracts that have therapeutic effects not only in OP, but are also effective in the treatment of oral and skeletal diseases, an institutional collaborative project between Showa University and Tokyo University of Marine Science and Technology was launched in 2010 [13]. Within this project, more than 400 bioactive herbal products were examined. After screening of the products by an osteoclast-formation-inhibition experiment utilizing RAW264.7 cells, 3 Chinese medical herbs, the root barks *Melia azedarach* (*M. azedarach;* commonly known as bead-tree or Cape lilac), *Corydalis turtschaninovii* (*C. turtschaninovii; *crested lark), and *Cynanchum atratum* (*C. atratum*; swallowwort) were chosen for further investigation. Although water or ethanol extracts of the roots were reported to contain biologically-active chemicals [14-19], the main compounds and precise mechanisms for the pharmacological effects of the extracts are unknown. In the present study, we reveal that these herbal extracts not only induce apoptosis of mature OCs, but also increase differentiation of OBs and chondrocytes *in vitro*. These findings suggest the feasibility of the use of these herbal extracts as novel therapeutics in OP.

## Methods

### Preparation of the root bark and BP

Approximately 400 kinds of dry herbal roots, including *M. azedarach, C. turtschaninovii* and *C. atratum*, were imported from China. The plant materials were formally surveyed and identified by Laboratory of Nutraceuticals and Functional Foods Science, Graduate School of Marine Science and Technology. The dry powdered roots (100 g) were extracted and concentrated to 1 mg/ml under reduced pressure as described previously [6]. Alendronate (AD; Wako Pure Chemical Industries, Osaka, Japan) was used as a BP control, and added at a final concentration of 0.01 to 100 μM into the culture medium.

### Cell culture

RAW264.7 cells (mouse monocytes) were cultured and allowed to differentiate into OCs, as described previously [20]. MC3T3E1 cells (mouse osteoblastic cells) were cultured in α-minimum essential medium (MEM) supplemented with 10% fetal bovine serum (FBS) and Osteoblast-Inducer Reagent, a cocktail of L-ascorbic acid, dexamethasone and β-glycerophosphoric acid, (Takara Bio, Shiga, Japan), and ATDC5 cells (mouse chondrosarcoma cells) were cultured in Dulbecco’s modified Eagle’s medium nutrient mixture F-12 Ham (DMEM/F-12) supplemented with 10% FBS and Insulin-Transferrin-Sodium selenite Supplement (Roche Diagnostics, Indianapolis, IN). Normal mouse bone marrow (MBM) cells from 8- to 9-wk-old female ICR mice were purchased from Takara Bio, and grown in RPMI 1640 medium supplemented with 10% FBS, according to the manufacturer’s protocol. All cells were grown at 37°C, 5% CO_2_ and 100% humidity.

### Histochemistry

Cells were seeded at a density of 3 × 10^3^ cells/ well in 48-well cell culture plates and permitted to grow to maturation for 3 (for RAW264.7 and MBM cells) or 7 days (for MC3T3 and ATDC5 cells) as described above. Thereafter, the herbal extracts (1, 10 and 100 μg/ml) or AD were added to the medium (0.5 ml/ well). After 3 days, the cells were stained for tartrate-resistant acid phosphatase (TRAP) [20] and alkaline phosphatase (ALPase) activity [21] and also stained with crystal violet [22], toluidine blue [22] and alizarine red [23], as described previously, with slight modification.

### Cell viability and apoptosis assays

For 3-[4, 5-dimethylthiazol-2-yl]-2, 5-diphenyltetrazolium bromide (MTT) assay, the cells were seeded at a density of 1 × 10^3^ cells/ well in 96-well cell culture plates (100 μl medium in each well). Once the cells were differentiated, herbal extracts or BP were added as described above, and the MTT assay was performed as described previously [24]. The activities of caspase 3/7, 8 and 9 were measured by Caspase-Glo (Promega, Madison, WI) and GloMax-Multi + Detection System (Promega), according to the manufacturer’s protocol. Genomic DNA fragmentation was investigated using a commercial kit (Apopladder EX, Takara), according to the manufacturer’s protocol.

### Western blotting analysis

Total cellular protein was prepared as described previously [25], protein concentration was measured by Quick Start Bradford Reagent (Bio-rad, Hercules, CA) using bovine serum albumin as a standard, and aliquots were stored at -80°C until use. Twenty micrograms of protein was subjected to sodium dodecyl sulfate polyacrylamide electrophoresis (SDS-PAGE) in 4- 20% gradient gel (Bio-Rad), and the blot was transferred onto polyvinylidene difluoride membrane (Life Technologies, Carlsbad, CA). Blocking, incubation with primary and horseradish peroxidase-conjugated secondary antibodies and washing of the blots were carried out as previously described [25]. Subsequently, the signal was visualized using Amersham ECL Western Blotting Detection Reagents (GE Healthcare UK Ltd., Buckinghamshire, UK) and ChimiDoc XRS Plus ImageLab System (Bio-Rad). The primary and secondary antibodies were purchased from Cell Signaling Technology (Danvers, MA) and GE Healthcare, respectively.

### Biochemical assays

Cells were seeded at a density of 3 × 10^3^ cells/ well in 24-well cell culture plates and cultured as described above in the presence of herbal extracts or AD (1 ml medium/ well). Thereafter, cells were lysed with 0.3 ml of 0.02% Triton X-100 (Sigma Aldrich, St. Louis, MO) in physiological saline, sonicated, and stored at -80°C until use. DNA and sulfated glycosaminoglycan (GAG) content were measured as described previously [22], and ALPase activity was assayed with a commercial kit (pNPP Phospatase Assay Kit, BioAssay Systems, Hayward, CA). Calcium and PO_4_ content were also measured with commercial kits (Calcium C-test and Phosphor C-test, Wako). Meanwhile, the conditioned medium (CM) of cultured cells was collected, centrifuged at 1 × 10^4^*g* for 5 min at 4°C, concentrated with Amicon Ultra-0.5 ml 3 k (Merck Millipore, Billerica, MA), and the resulting aliquot (25 μl) was subjected to enzyme-linked immunosorbent assay (ELISA) for mouse osteocalcin using a commercial kit (Mouse Osteocalcin EIA Kit, Biomedical Technologies, Stoughton, MA). Osteocalcin content and activity were normalized to DNA content in the cell layer lysate.

### RNA isolation and real-time PCR analysis

Cells were seeded in a 24 well culture plate, and cultured as described above (1 ml medium/ well). Total RNA was purified using a commercial kit (NucleoSpin RNA II, Macherey-Nagel, Düren, Germany), and single-strand cDNA was reverse-transcribed from a 100 ng aliquot of total RNA using a random nonamer and MV Reverse Transcription XL (Takara Bio) according to the manufacturer’s protocol. Real-time PCR was performed with the SYBR green system (MyiQ2; Bio-Rad). One nanogram of each cDNA was used as a template, under conditions of 1 nM of each primer pair and 10 μl of the 2× iQ SYBR Green Supermix (Bio-Rad) in a total volume of 20 μl. The primer sequences are as shown in Table 1. Statistical analysis was performed using Bio-Rad iQ5 analysis software. Gene expression was first normalized to β-actin within each sample group and the fold change in gene expression was calculated using the 2^-ΔΔCt^ method.

**Table 1:** The genes, primer sequence for sense (upper row) and anti-sense (lower row) strands references and GenBank accession numbers utilized for real-time PCR are shown

**Gene**	**Primer sequence**	**Reference**	**GenBank accession No**
ALPase	5′-TGACCTTCTCTCCTCCATCC-35′	[26]	NM_007431
	5′-CTTCCTGGGAGTCTCATCCT-3′		
Osteocalcin	5′-TGCTTGTGACGAGCTATCAG-3′	[26]	NM_007541
	5′-GAGGACAGGGAGGATCAAGT-3′		
Col II α1	5′-ACTGGTAAGTGGGGCAAGAC-3′	[26]	NM_031163
	5′-CCACACCAAATTCCTGTTCA-3′		
Aggrecan	5′-AGGACCTGGTAGTGCGAGTG-3′	[27]	NM_007424
	5′-GCGTGTGGCGAAGAA-3′		
β-actin	5′-AGATGTGGATCAGCAAGCAG-3′	[26]	NM_007393
	5′-GCGCAAGTTAGGTTTTGTC-3′		

### Statistical analysis

Unless otherwise specified, all experiments were repeated at least three times, and similar results were obtained in the repeated experiments. The two-tailed, unpaired Student’s *t*-test was used for analysis. Data are expressed as means ± standard deviation of triplicate data. A *P*-value < 0.05 was considered significant.

## Results

### Assessment of optimal dosage of alendronate used as an apoptosis-inducible control

**Figure 1:**
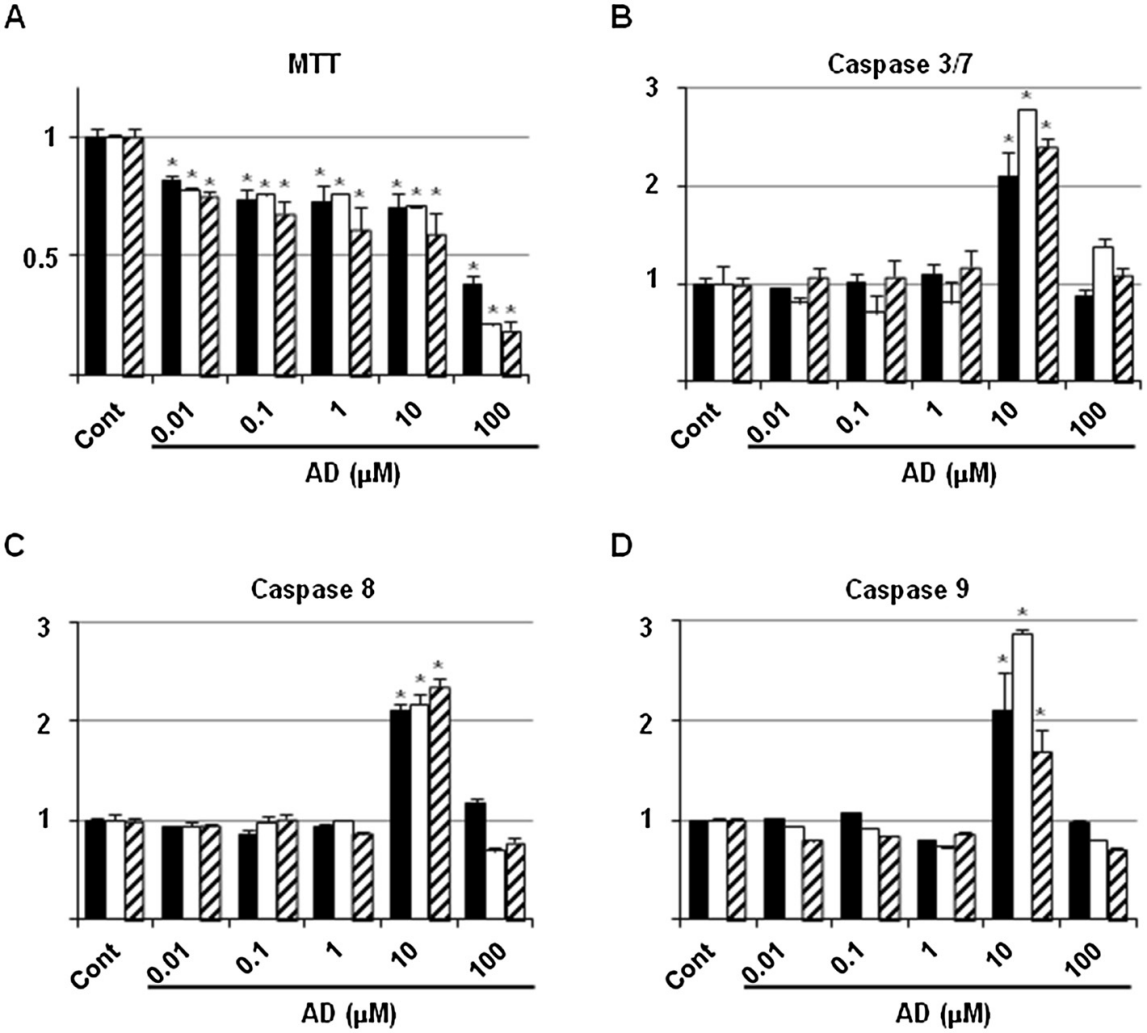
**Optimal dosage of AD used as an apoptosis-inducible positive control.** RAW264.7 (closed box), MC3T3E1 (open box) and ATDC5 (shaded box) cells were seeded at a density of 1×10^4^ cells/well in 96-well tissue culture plates, and grown in the absence (Cont) or presence of 0.01 to 100 μM of AD. After 24 h, cells were subjected to MTT **(A)**, and caspase 3/7 **(B)**, 8 **(C)** and 9 **(D)** assays. Data are means ± standard deviation of the values from 3 sets of cultures. *, *p* < 0.05 *vs* each control.

Before commencement of the final screening of herbal extracts, the optimal dosage of AD was examined in the three cell lines (RAW264.7, MC3T3E1 and ATDC5 cells) that were subjected to final screening (Figure 1). At low concentrations (0.01 to 1 μM), AD had negligible effects on either cell viability or caspase activities. A dose of 10 μM of AD, however, effectively reduced cell viability represented by MTT assay, and induced activation of caspases, indicating that all of the cells underwent apoptosis. Furthermore, 100 μM of AD decreased not only cell viability, but also caspase activities. These results indicated that a concentration greater than 10 μM induces not only apoptosis, but also necrosis due to cytotoxicity, resulting in relative decrease both of MTT and of caspase activities. Based on these data, we chose to use 10 μM of AD as an apoptosis-inducible control in the following experiments.

### *In vitro* screening of herbal extracts utilizing TRAP staining in OCs

**Figure 2:**
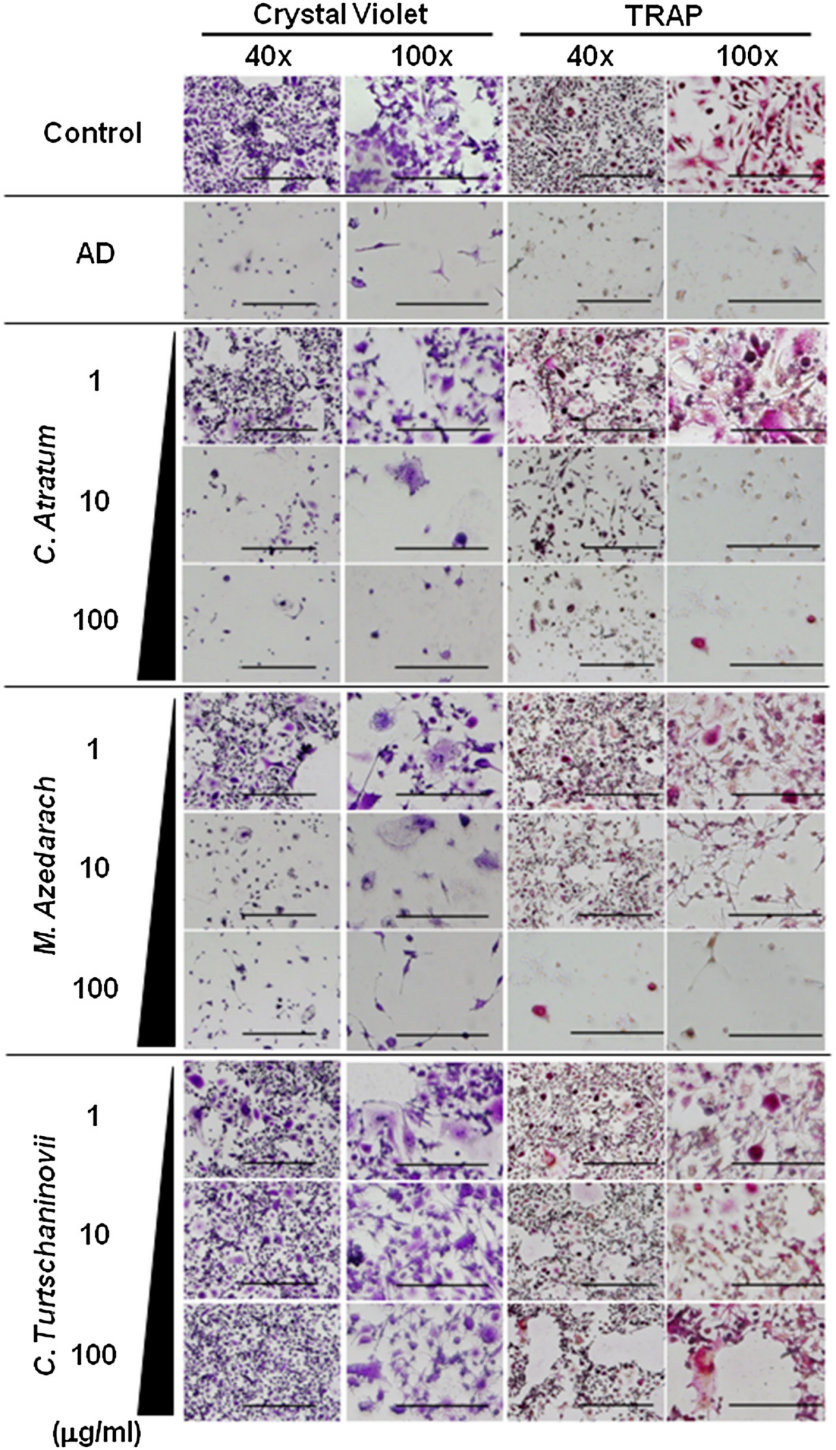
**Inhibition of mutation and induction of cell death in OCs by incubation with herbal extracts.** RAW 264.7 cells were seeded and allowed to differentiate into OCs. The cells were then cultured in the absence (Control) or presence of 10 μM AD, or 1, 10 and 100 μg/ml *C. atratum*, *M. azedarach* and *C. turtschaninovii* extracts. After 3 days, cells were stained with crystal violet and TRAP and examined with a microscope at a magnification of 40× and 100×. Bar, 1 mm (40×) and 100 μm (100×).

As described in our recent study [13], more than 400 bioactive herbal extracts were subjected to *in vitro* preliminary screening in which differentiated OCs from RAW264.7 cells were cultured in the presence of the extracts for 3 days. During this screening procedure, extracts that demonstrated growth-inhibitory or apoptosis-inducing effects on OCs, which were induced from RAW264.7 cells, were selected. These selected extracts were subjected to secondary screening whereby differentiated OBs from MC3T3E1 cells and chondrocytes from ATDC5 cells were cultured in the presence of the extracts for 3 days. Finally, extracts that did not induce cell death were selected. As a result, 3 herbal extracts from the root bark of 1) *M. azedarach,* 2) *C. turtschaninovii* and 3) *C. atratum* were determined to be adequate for the present study and were utilized in the following experiments. Figure 2 shows the effects of these extracts and AD on RAW264.7 cells by crystal violet and TRAP staining. In cells treated with the control, TRAP-positive, multinuclear and giant cells, which reflect a typical phenotype of OCs, were observed. Treatment with the herbal extracts at a concentration of 1 μg/ml slightly decreased the number of total and OC-induced cells. This growth-inhibitory effect was more pronounced in cultures treated with 10 μg/ml or more of the herbal extracts, and occurred in a dose-dependent manner. Moreover, at a concentration of 100 μg/ml, OC-like cells were barely detectable by TRAP staining, and cell number was drastically reduced as shown by crystal violet staining. These results were also observed in cell cultures to which AD was added.

### The herbal extracts induce apoptosis in OCs via activation of caspases

**Figure 3:**
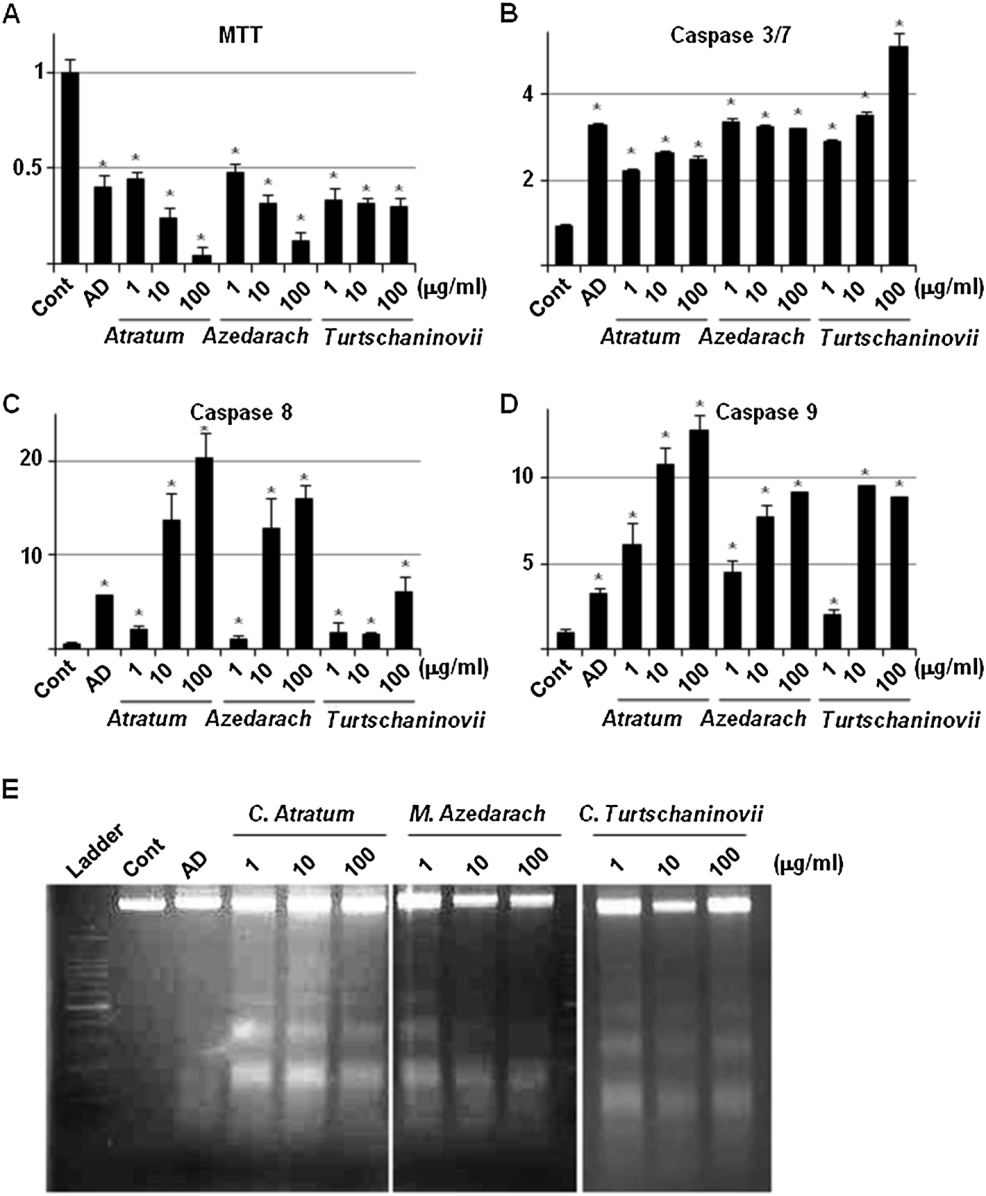
**Apoptotic effects of the herbal extracts on OCs.** RAW264.7 cells were seeded in 96-well cell culture plates and allowed to differentiate into OCs. Cells were cultured in the absence (Control) or presence of 10 μM AD, or 1, 10 and 100 μg/ml *C. atratum* (*Atratum*) *M. azedarach* (*Azedarach*) and *C. turtschaninovii* (*Turtschaninovii*) extracts. After 3 days, the cells were subjected to MTT **(A)**, caspase 3/7 **(B)**, 8 **(C)** and 9 **(D)** assays. The genomic DNA fragmentation assay **(E)** was carried out using a commercial kit, according to the manufacturer’s protocol. In the left adjacent lane, a 100 bp DNA ladder (Takara) was applied; bold bans indicate 1000 and 500 bp. Data in *panel* a to d are means ± standard deviations of 3 cultures, and each mean is valued relatively as “1.” *, *p* < 0.05 *vs* control.

We investigated the mechanism by which the extracts decrease cell number of OCs. Results from the MTT assay (Figure 3A) showed more accurately that all of the extracts decreased cell viability more than by treatment with AD. The caspase assays (Figure 3B, C and D) revealed that this effect on cell viability was dependent on up-regulation of the activities of caspase 3/7, 8 and 9 in control and extract-treated cells. The concentration at which maximal effects were observed as well as the specific caspase that demonstrated the greatest up-regulation in activity, however, differed for each extract. Treatment with the extracts also resulted in DNA fragmentation (Figure 3E) suggesting that these herbal extracts can induce not only growth inhibition, but also apoptosis in induced OCs.

**Figure 4:**
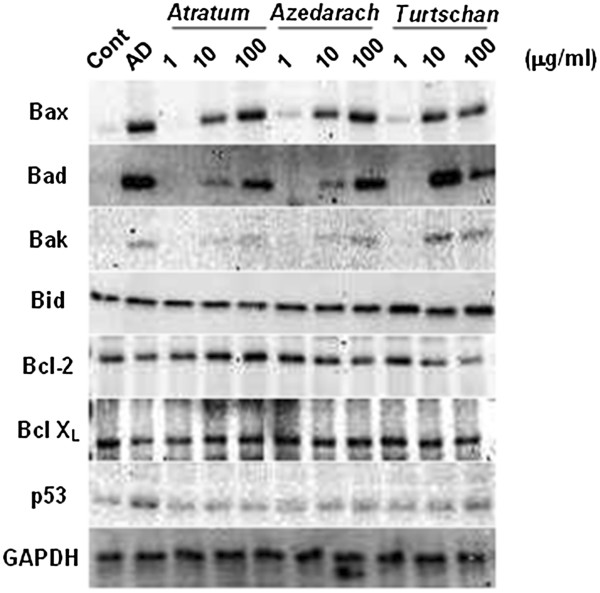
**Modulation of apoptosis-related proteins by stimulation with herbal extracts.** RAW264.7 cells were allowed to differentiate into OCs. a. The cells were seeded at a density of 1×10^5^ cells /well in 6-well culture plates, and allowed to differentiate into osteoclasts by the addition of RANKL. Thereafter, the cells were cultured in the absence (Cont) or presence of 10 μM AD, or 1, 10 and 100 μg/ml *C. atratum* (*Atratum*) *M. azedarach* (*Azedarach*) and *C. turtschaninovii* (*Turtschan*) extracts. After 3 days, total cellular protein was collected, and 20 μg of the aliquot was subjected to Western Blotting analysis for Bax, Bad, Bak, Bid Bcl-2, Bcl-X_L_, p53 and GAPDH, sequentially.

Further investigation into the apoptotic effects of the extracts was carried out by Western blot analysis for several apoptosis-related molecules (Figure 4). Treatment with the herbal extracts resulted in a robust increase in the mitochondria-related pro-apoptotic proteins Bax, Bad and Bak, except for Bid, whereas changes in the pro-survival proteins, Bcl-2 and Bcl-X_L_ were not observed. In contrast, protein expression of p53 was increased only by AD. These results suggest that the apoptotic effects of the herbal extracts might be regulated by up-regulation of a mitochondria-related apoptotic pathway.

### The herbal extracts do not enhance proliferation but induce differentiation of OBs

**Figure 5:**
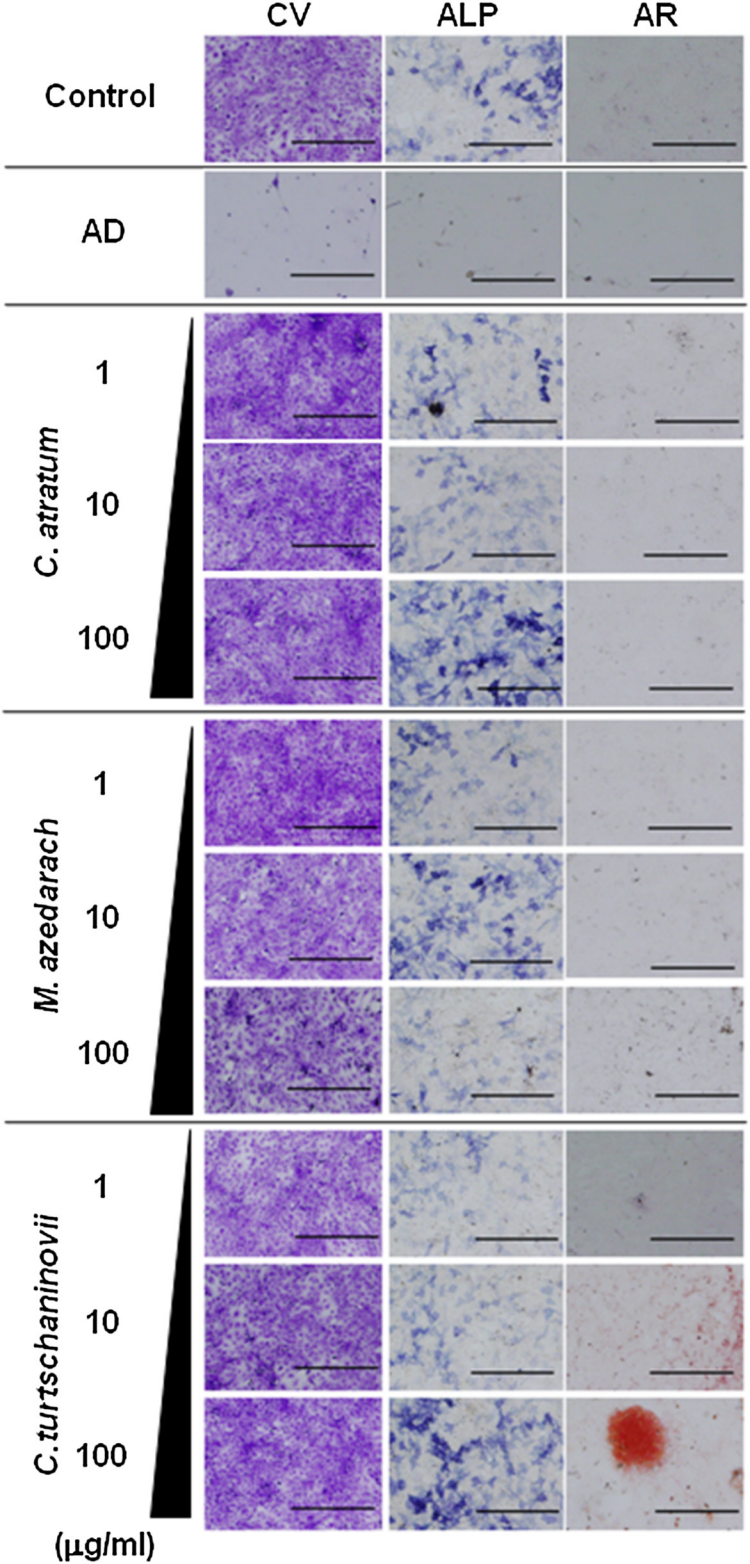
**Histochemical analysis of OB-specific phenotypes induced by herbal extracts.** MC3T3E1 cells were seeded and allowed to differentiate into mature OBs. Then, the cells were then cultured in the absence (Control) or presence of 10 μM AD, or 1, 10 and 100 μg/ml *C. atratum*, *M. azedarach* and *C. turtschaninovii* extracts. After 3 days, cells were subjected to crystal violet (CV), ALPase activity (ALP) and alizarin red (AR) staining, and examined under a microscope. Bar, 100 μm.

**Figure 6:**
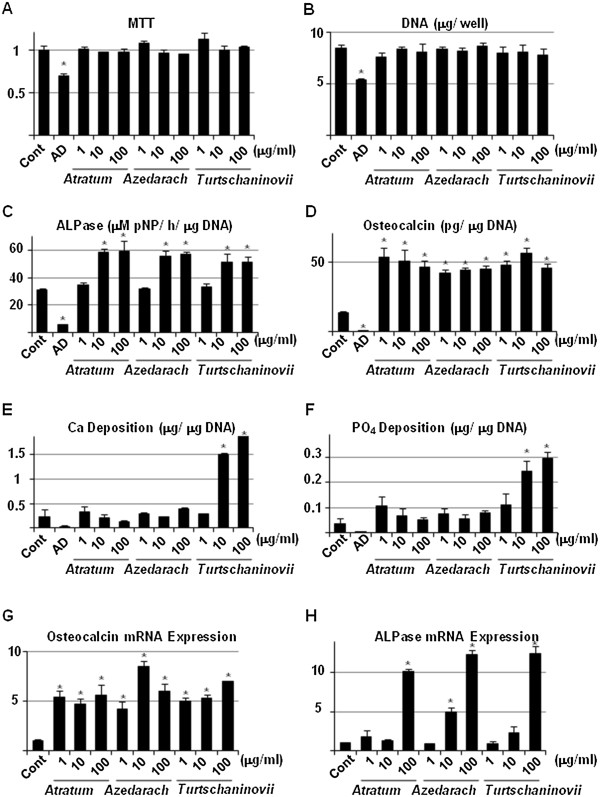
**Biochemical analysis and gene expression of OB-specific phenotypes induced by herbal extracts. A**, MC3T3E1 cells were seeded in 96-well cell culture plates and allowed to differentiate into mature OBs. Then, cells were then cultured in the absence (Control) or presence of 10 μM AD, or 1, 10 and 100 μg/ml *C. atratum* (*Atratum*) *M. azedarach* (*Azedarach*) and *C. turtschaninovii* (*Turtschaninovii*) extracts. After 3 days, the cells were subjected to MTT assay. **B**, **C**, **D**, **E** and **F**, MC3T3E1 cells were seeded in 24-well cell culture plates and allowed to differentiate into mature OBs. Thereafter, cells were cultured in the absence (Control) or presence of 10 μM AD, or 1, 10 and 100 μg/ml *C. atratum* (*Atratum*) *M. azedarach* (*Azedarach*) and *C. turtschaninovii* (*Turtschaninovii*) extracts. After 7 days, the cell layer was lysed with 300 μl of 0.02% Triton-X 100 in saline. The aliquot was then subjected to DNA **(B)**, ALPase activity **(C)**, Ca **(E)**, PO_4_**(F)** measurement. The CM was also concentrated and subjected to ELISA for osteocalcin **(D)**. Data are means ± standard deviation of 3 sets of cultures and each value (except for DNA) is standardized to DNA content (μg) in the cell layer. G and H, MC3T3E1 cells were seeded and cultured, as described above. After 7 days, total cellular RNA was purified, and real-time RT-PCR for osteocalcin **(G)** and ALPase **(H)** was carried out. Data are means ± standard deviation of 3 sets of cultures and each value is standardized to β-actin. *, *p* < 0.05 *vs* control.

Since the herbal extracts caused drastic growth-inhibitory and apoptosis-inducible effects in induced OCs, we investigated whether they would have the same effects on MC3T3E1 cells, an OB cell line (Figures 5 and 6). We did not observe effects of the extracts on cell proliferation, as demonstrated by crystal violet staining (Figure 5, CV), MTT assay (Figure 6A) and DNA measurement (Figure 5B). AD inhibited growth and differentiation of OB. Conversely, treatment with the extracts resulted in increased ALPase activity (Figure 5, ALP and Figure 6C) and osteocalcin secretion (Figure 6D) that are markers of differentiation in OBs. Furthermore, a concentration of 100 μg/ml of the extract of C. *atratum* enhanced calcification of the extra-cellular matrix (ECM), as shown by alizarin red staining (Figure 5, AR), in addition to Ca and PO_4_ assays (Figure 6E and F). Real-time RT-PCR (Figure 6G and H) revealed that addition of the extracts increased mRNA of osteocalcin and ALPase, confirming the results of histochemical and biochemical assays. In particular, mRNA expression of ALPase was increased in a dose-dependent manner by treatment with the extracts, whereas that of osteocalcin did not always do. Thus, these results reveal the potential ability of the herbal extracts to act as therapeutic agents in OP.

### The herbal extracts increase ALPase activity of chondrocytes, but do not affect synthesis of cartilage-specific ECM

**Figure 7:**
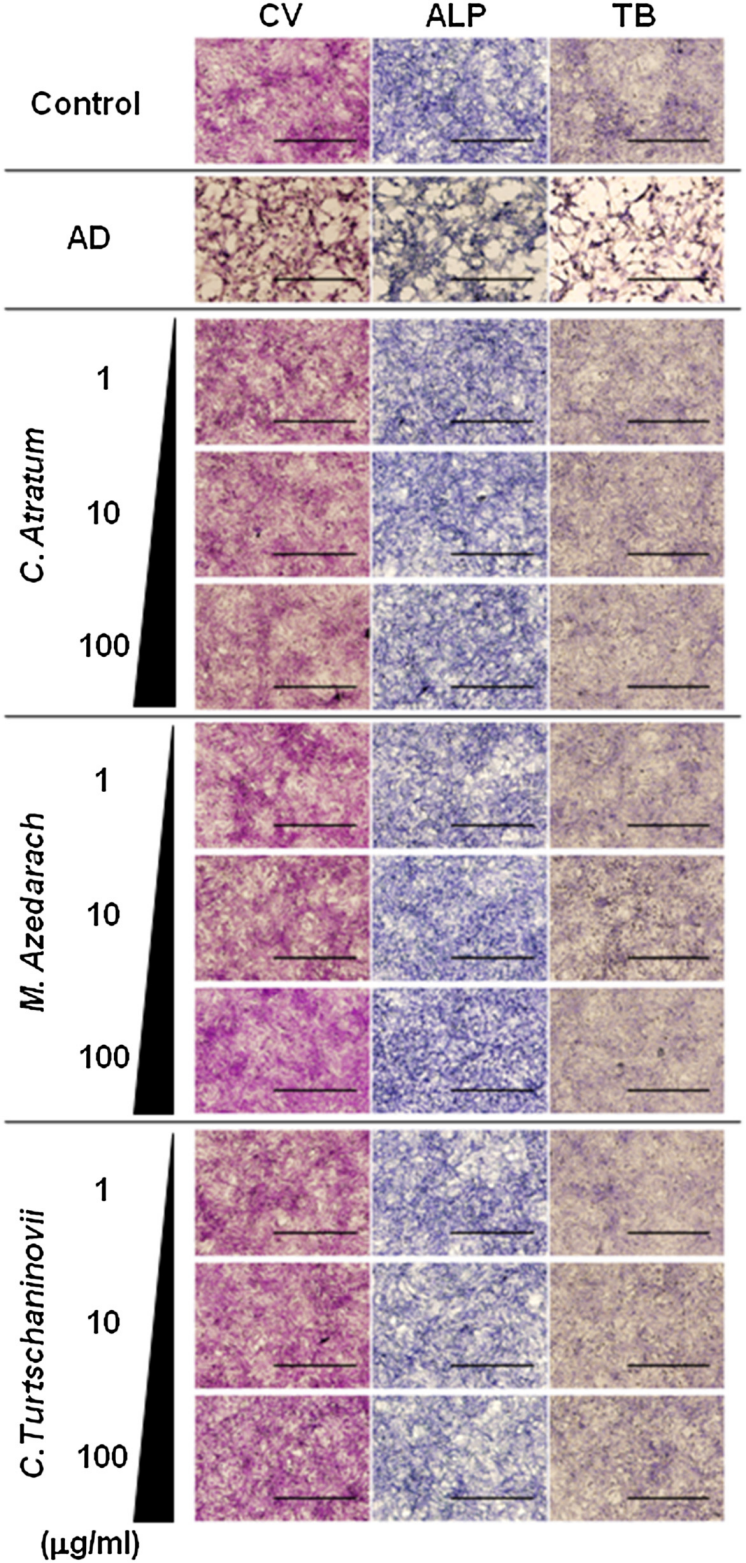
**Histochemical analysis of chondrocyte-specific phenotypes induced by herbal extracts.** ATDC5 cells were seeded and allowed to differentiate. Thereafter, cells were cultured in the absence (Control) or presence of 10 μM AD, or 1, 10 and 100 μg/ml of *C. atratum*, *M. azedarach* and *C. turtschaninovii* extracts. After 3 days, cells were subjected to crystal violet (CV), ALPase activity (ALP) and toluidine blue (TB) staining, and examined under a microscope. Bar, 100 μm.

**Figure 8:**
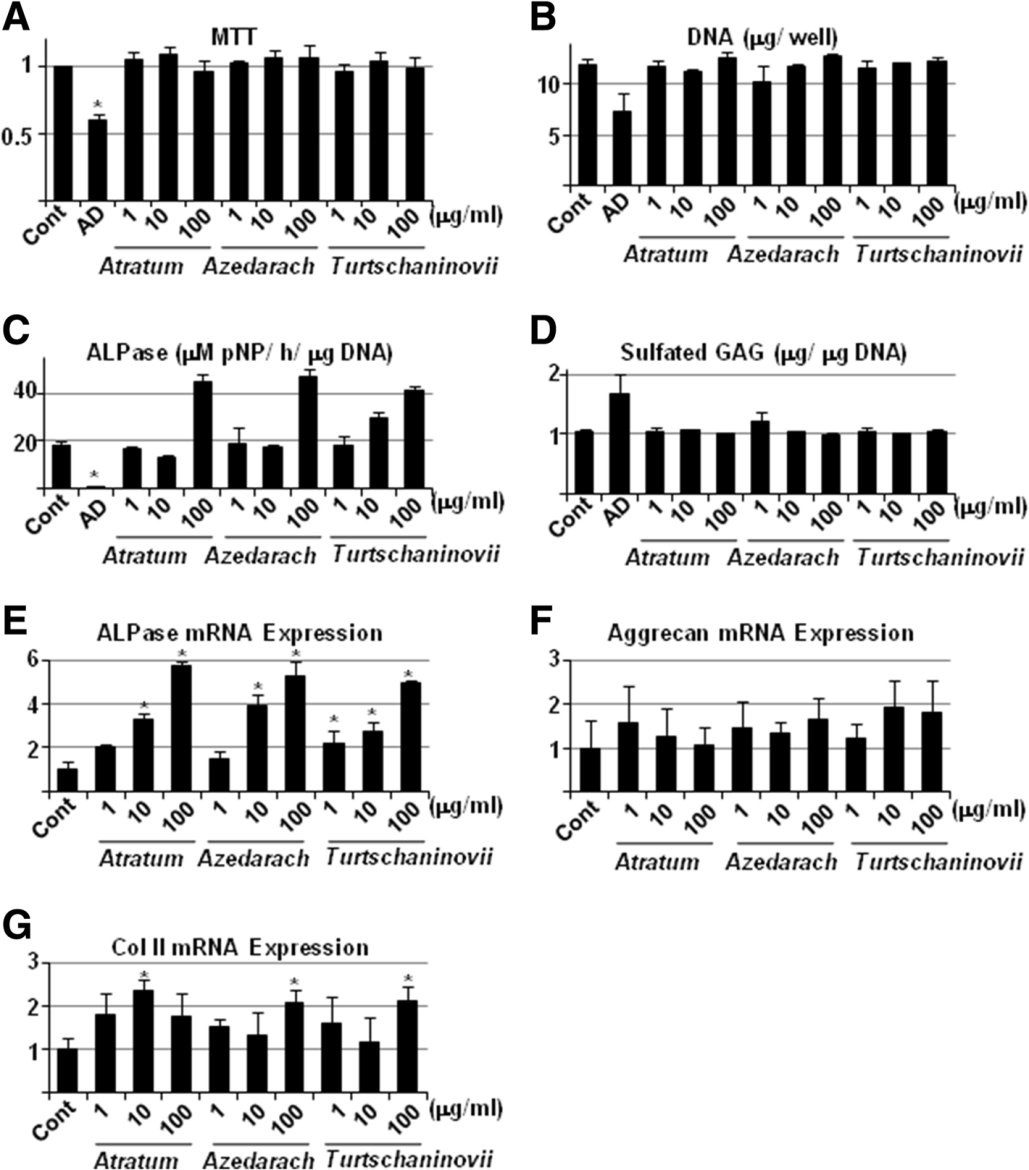
**Biochemical analysis and gene expression of chondrocyte-specific phenotypes induced by herbal extracts. A**, ATDC5 cells were seeded in 96-well cell culture plates and allowed to differentiate. Thereafter, cells were cultured in the absence (Control) or presence of 10 μM AD, or 1, 10 and 100 μg/ml *C. atratum* (*Atratum*) *M. azedarach* (*Azedarach*) and *C. turtschaninovii* (*Turtschaninovii*) extracts. After 3 days, cells were subjected to MTT assay. **B**, **C** and **D**, ATDC5 cells were seeded in 24-well cell culture plates and allowed to differentiate. Thereafter, cells were cultured in the absence (Control) or presence of 10 μM AD, or 1, 10 and 100 μg/ml *C. atratum* (*Atratum*) *M. azedarach* (*Azedarach*) and *C. turtschaninovii* (*Turtschaninovii*) extracts. After 7 days, the cell layer was lysed with 300 μl of 0.02% Triton-X 100 in saline. The aliquot was then subjected to DNA **(B)**, ALPase activity **(C)** and sulfated GAG **(D)** measurement. Data are means ± standard deviation of 3 sets of cultures, and each value (except for DNA) is standardized to DNA content (μg) in the cell layer. **E**, **F** and **G**, ATDC5 cells were seeded and cultured, as described above, and then total cellular RNA was purified, and real-time RT-PCR for ALPase **(E)**, aggrecan **(F)** and type II collagen **(G)** was carried out. Data are means ± standard deviation of 3 sets of cultures and each value is standardized to β-actin. *, *p* < 0.05 *vs* control.

Our next step was to determine the biological effects of the herbal extracts on chondrocytes. ATDC5 cells were cultured in the presence of the extracts for 1 week, and then subjected to histochemical staining (Figure 7) and biochemical assays (Figure 8). We did not observe significant effects of the herbal extracts on cell proliferation as shown by crystal violet staining (Figure 7, CV), MTT assay (Figure 8A) and DNA measurement (Figure 8B), although AD was able to decrease cell viability. Similar to the results observed in MC3T3E1 cells. Although histochemical staining (Figure 7, ALP) showed little difference, biochemical measurement (Figure 8C) was capable of representing the up-regulated ALPase activity by all of the extracts at a concentration of 1 μg/ml, and stronger effects were observed with concentrations greater than 10 μg/ml. Nonetheless, accumulation of sulfated GAG (Figure 7, TB and Figure 8D) in the cartilage ECM was similar in the presence or absence of the extracts. For more detailed assessment of effects on ECM synthesis, real-time RT-PCR for aggrecan core protein (Figure 8E) and type II collagen (Figure 8F) was carried out. The results not only corroborated those from cytochemistry and bioassays, but also clarified the effects of the extracts, particularly on ALPase activity. Although RT-PCR for type X collagen was also carried out, the threshold cycle (Ct) in each condition was more than 45, indicating rare gene expression (data not shown). Therefore, in agreement with the results from the bioassay, the herbal extracts showed little effect (*p* > 0.05) on gene expression. These data suggest that the herbal extracts may play a role in increasing direct ossification of cartilage tissue but not maturation.

### The herbal extracts induce osteoblastic, but not osteoclastic differentiation in primary bone marrow cells

**Figure 9:**
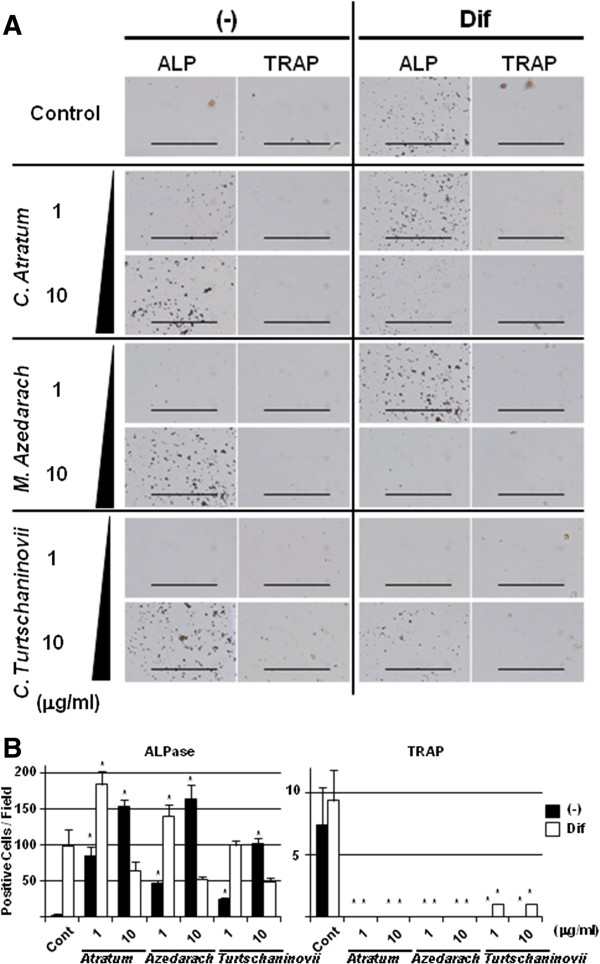
**Histochemical analysis of primary MBM cells for differentiation induced by herbal extracts.** MBM cells were seeded at a density of 1.5×10^4^ cells /well in 6-well tissue culture plates, and cultured with (Dif) or without (-) commercially available Osteoblast-Inducer Reagent, in the absence (Control) or presence of 1 and 10 μg/ml *C. atratum*, *M. azedarach,* and *C. turtschaninovii* extracts. After 7 d, the cells were subjected to ALPase activity (ALP) or TRAP staining. **A**, The cells were examined by a microscope, and photographed. Bar, 500 μm. **B**, The number of positive cells was counted by a microscope, and means ± standard deviation of 3 individual fields were shown. *, *p* < 0.05 *vs* each control.

In the above experiments, three cell lines were used. Finally, we investigated whether the effects of the herbal extracts are observed in a primary mouse bone marrow (MBM) cells (Figure 9). Seven days after seeding, few cells (< 50%) survived in all conditions. However, the live cells differentiated into two populations, one was ALPase-positive; the other was TRAP-positive. This observation was more pronounced in the presence of Osteoblast-Inducer Reagent, particularly in low dosage of the extracts. The addition of herbal extracts resulted in an increased number of ALPase-positive cells. In contrast, no TRAP-positive cells were detected, even in the presence of Osteoblast-Inducer Reagent. These results indicate that the extracts induced differentiation of bone marrow stem cells into OBs, but not OCs, and strongly suggest the clinical utility of these herbal extracts as a therapeutic against OP.

## Discussion

A number of basic and clinical studies investigating chemical treatment for OP, including parathyroid hormone (PTH), vitamin D_3_ and selective estrogen receptor modulator (SERM), have been reported. Currently, BPs are widely used as a therapeutic medicine for OP, as well as bone-metastatic cancers, since they effectively inhibit bone resorption. Despite great pharmacological and clinical advantages of BPs, however, serious side effects, such as renal failure and BRONJ, have also been reported [4]. This suggests an urgent need for the identification and development of novel medicines. Botanical therapeutics are traditional medicines such as Chinese herbal medicines. The therapeutic effects of a variety of herbal extracts have been studied and reported worldwide. Among these, one of the most investigated herbal extracts is curcumin (reviewed in ref. [28]), which is isolated from the rhizome of *Curcuma longa* (commonly known as turmeric). Curcumin has been reported to have anti-cancer, anti-viral, anti-arthritic, anti-amyloid, anti-oxidant, and anti-inflammatory properties, and is considered a potential therapeutic agent in the prevention and/or treatment of various malignant diseases, arthritis, allergies, Alzheimer’s disease, inflammation and OP [9-12]. This accumulation of evidence encouraged us to explore the use of herbs other than *C. longa* for their potential therapeutic effects in OP, and hence an institutional collaborative project was commenced [13].

During bone remodeling [29], bone resorption by OCs occurs prior to bone formation by OBs. It has been suggested that suppression of proliferation and maturation of OCs prevents excess bone loss. To this end, more than 400 bioactive herbal products were subjected to a preliminary screening utilizing RAW264.7 cells, and we subsequently narrowed these candidates down to three: *M. azedarach, C. turtschaninovii* and *C. atratum*. The bark of *M. azedarach* has been utilized as a therapeutic medicine for tinea imbricata in the Chinese pharmacopoeia [18,19]. Methanolic extract of *C. turtschaninovii* has been reported to have anti-allergic effects [15], and is used in traditional Chinese medicine in the treatment of gastric and duodenal ulcers, cardiac arrhythmic disease, rheumatism, and dysmenorrhea [14]. Finally, the root bark extract of *C. atratum* has been used as an anti-febrile and diuretic [16], and has been reported to show anti-acetylcholinesterase and anti-amnesic activities *in vivo* in mice [17].

In addition to previous reports on pharmacological availability, we showed in the present study that these extracts are capable not only of suppressing proliferation and/or maturation of OCs (Figure 2), but also of inducing cell death (apoptosis) by increasing caspase activity (Figure 3). The results of biochemical assays showed inconsistencies with those of hystochemistry in part. However, it is supposed that the former represents more accurate results than the later does, since histochemical staining often detects a non-specific artifact, and thereby, shows inconsistent results. It is important to note that an increase in the mitochondrial pro-apoptotic/pro-survival protein ratio is required for apoptosis in various cells, including OCs [30]. All of the extracts increased expression of Bax, Bad and Bak, whereas the effects on expression of Bcl-2 and Bcl-X_L_ differed for each extract (Figure 4). On the otherhand, AD increased p53 protein, as well as Bax, Bad and Bak. In any case, the Bcl-2 pathway and subsequent activation of caspase is involved in the apoptotic effects observed with these compounds, and interestingly, it is suggested thay the apoptotic signal pathway might be different from that by BPs.

Since anti-OP therapeutics are required to have minimal effects in reducing bone formation, we investigated the effects of the herbal extracts on proliferation and differentiation of OBs (Figures 5 and 6) and chondrocytes (Figures 7 and 8) cell lines. All of the extracts exhibited positive effects on partial, but not terminal, maturation of OBs and chondrocytes, suggesting that these compounds satisfy the requirements for therapeutics used in OP. Finally, the extracts demonstrated the ability to induce differentiation of OBs, but not OCs, from primary MBM cells, reinforcing the effects of Osteoblast-Inducer Reagent, albeit the effect was likely to be saturated at the high dosage (10 μg/ml) of those extracts (Figure 9). The results from the present study imply that we have successfully, at least in part, uncovered a novel potential activity of these extracts to be used as medicines for OP. We are of course aware that further investigation, such as isolation and analysis of bioactive chemicals, detailed molecular and cellular experiments *in vitro*, and pre-clinical studies *in vivo*, is required in order to ensure that there are not serious side effects associated with the use of the herbal extracts, as has been reported with chemically synthesized medicines. Indeed, several of these studies are currently underway, and our findings will be reported in the near future.

## Conclusions

In the present study, we have successfully uncovered a novel potential activity of three Chinese medical herbal extracts from the root barks of *M. azedarach*, *C. turtschaninovii*, and *C. atratum* to be used as medicines for OP. All of the extracts showed capabilities of inducing OCs to undergo apoptosis, OBs and chondrocyte to differentiate, but not to grow. Moreover, the extracts induced osteoblastic, but not osteoclastic differentiation in primary MBM cells. In conclusion, these findings suggest the feasibility of the use of these herbal extracts as novel therapeutics in OP.

## Abbreviations

OP: Osteoporosis; BP: Bisphosphonate; BRONJ: Bisphosphonate-related osteonecrosis of the jaw; OC: Osteoclast; OB: Osteoblast; M. azedarach: *Melia azedarach*; C. turtschaninovii: *Corydalis turtschaninovii*; C. atratum: *Cynanchum atratum*; AD: Alendronate; MEM: Minimum essential medium; FBS: Fetal bovine serum; DMEM/F-12: Dulbecco’s modified Eagle’s medium nutrient mixture F-12 Ham; MBM: Mouse bone marrow; TRAP: Tartrate-resistant acid phosphatase; ALP: Alkaline phosphatase; MTT: 3-[4, 5-dimethylthiazol-2-yl]-2, 5-diphenyltetrazolium bromide; SDS-PAGE: Sodium dodecyl sulfate polyacrylamide electrophoresis; GAG: Glycosaminoglycan CM, conditioned medium; ELISA: Enzyme-linked immunosorbent assay; ECM: Extra-cellular matrix; PTH: Parathyroid hormone; SERM: Selective estrogen receptor modulator.

## Competing interests

The authors declare that they have no financial competing interests.

## Authors’ contributions

YM significantly contributed to and performed the present study, applied for the grant supporting the study, prepared the figures, and wrote the manuscript. SK applied for the grant supporting the study, and helped to draft the figures and manuscript. CL, SB and AK performed histochemical and biochemical assays. TK prepared the materials and purified the herbal extracts. KY participated in the design and coordination of the study. SS conceived the idea, applied for the grant supporting the study, and proofread the figure and manuscript. All authors read and approved the final manuscript.

## Pre-publication history

The pre-publication history for this paper can be accessed here:

http://www.biomedcentral.com/1472-6882/14/29/prepub
